# Atypical Skull-Base Osteomyelitis: Comprehensive Review and Multidisciplinary Management Viewpoints

**DOI:** 10.3390/tropicalmed8050254

**Published:** 2023-04-28

**Authors:** Jure Urbančič, Domen Vozel, Saba Battelino, Roman Bošnjak, Barbara Kokošar Ulčar, Tadeja Matos, Matic Munda, Lea Papst, Nejc Steiner, Matej Vouk, Nina Zidar

**Affiliations:** 1 Department of Otorhinolaryngology and Cervicofacial Surgery, University Medical Centre Ljubljana, 1000 Ljubljana, Slovenia; 2 Faculty of Medicine, University of Ljubljana, 1000 Ljubljana, Slovenia; 3 Department of Neurosurgery, University Medical Centre Ljubljana, 1000 Ljubljana, Slovenia; 4 Department of Infectious Diseases, University Medical Centre Ljubljana, 1000 Ljubljana, Slovenia; 5 Institute of Microbiology and Immunology, Faculty of Medicine, University of Ljubljana, 1000 Ljubljana, Slovenia; 6 Department of Radiology, University Medical Centre Ljubljana, 1000 Ljubljana, Slovenia; 7 Institute of Pathology, Faculty of Medicine, University of Ljubljana, 1000 Ljubljana, Slovenia

**Keywords:** osteomyelitis, osteitis, aspergillosis, mucormycosis, clivus, sphenoid sinusitis, surgical endoscopy

## Abstract

Atypical skull-base osteomyelitis is a rare but fatal disease that usually involves infection of the ethmoid, sphenoid, occipital, or temporal bones that form the skull base. Unlike typical (so-called otogenic), atypical skull-base osteomyelitis has no otogenic cause. Instead, some authors call atypical skull-base osteomyelitis sinonasal, since the infection most often originates from the nose and paranasal sinuses. Diagnosing and treating this disease is challenging. To assist in managing atypical skull-base osteomyelitis, a review of the most recent literature, with patient cases and multidisciplinary perspectives from otolaryngologists, neurosurgeons, radiologists, infectious disease specialists, pathologists, and clinical microbiologists, is provided in this paper.

## 1. Introduction

Percival Pott first described osteomyelitis of the cranial bones in 1775 in a patient with a sub-pericranial abscess resulting from a frontal bone injury [1]. Later, it became known that the cause of such an infection was not an injury but the spread of infection from neighbouring structures, for example, paranasal sinuses. Meltzer and Kelemen first described skull-base osteomyelitis (SBO) in 1959 in patients with a burn injury and osteomyelitis of the external auditory canal [2]. Subsequently, it became known that SBO is not only the result of the progression of inflammation of the external auditory canal [3] but also inflammation of the face, nose, paranasal sinuses, oral cavity, and pharynx [4,5].

Atypical skull-base osteomyelitis (ASBO) is a rare but fatal disease and usually involves infection of the ethmoid, sphenoid, occipital, or temporal bones that make up the skull base. Unlike typical SBO, which is usually the result of advanced necrotising external otitis (so-called otogenic), ASBO does not have an otogenic cause. Some authors call ASBO sinonasal or non-otogenic SBO [4]. Other authors divide it into non-sino-rhino-otogenic and sino-rhino-otogenic, and the latter into SBO of the front (i.e., anterior), middle (i.e., central), and posterior cranial base (i.e., posterior SBO) [5].

Distinguishing between typical and ASBO is challenging, as the inflammation can involve the clivus in both types. In this case, they can be clinically distinguished, especially otoscopically, since symptoms and signs of inflammation of the external auditory canal or middle ear are usually present in typical SBO. The latter is also a better-known disease, and diagnosis is usually not challenging [6]. In contrast, diagnosing and treating ASBO is challenging.

This paper reviews the latest literature and the perspectives of an otorhinolaryngologist, neurosurgeon, radiologist, infectious disease specialist, pathologist, and clinical microbiologist managing ASBO.

## 2. Pathogenesis of Atypical Skull-Base Osteomyelitis

### 2.1. Causes and Routes of Disease Spread

ASBO can occur as a result of advanced or untreated infection of the deep tissues of the face, oral cavity, pharynx, or nasal and paranasal sinuses, usually the sphenoid (i.e., basisphenoid) and occipital bones (i.e., basiocciput) [4,7]. Rarely, the cause of the infection is hematogenous from a remote source, e.g., from the lung or spine [5,8].

The infection spreads along the soft tissues at the skull base, and when it invades the Haversian canals, it also begins to spread along the cancellous bone. Due to the spread of the infection, neurovascular structures are affected along their extracranial course, through their foramina at the skull base, and intracranially. Therefore, knowledge of the precise surgical anatomy of the skull base is necessary to understand the clinical picture of ASBO.

The most common cause of ASBO is an advanced paranasal sinus infection. From the sphenoid sinus, which is the centre of the skull base, the infection can spread in all directions, i.e., anterior, middle, or posterior cranial fossa, orbit, and adjacent paranasal sinuses. From the ethmoid cells, the infection first spreads to the adjacent paranasal sinuses (frontal, maxillary, and sphenoid sinus) and then across the borders of the paranasal sinuses to the orbit and, above all, to the intracranial space. From the maxillary sinus, the infection (e.g., odontogenic maxillary sinusitis) spreads through the pterygopalatine and infratemporal fossa into the middle cranial fossa, orbit, sphenoid sinus, and through the ostiomeatal complex, into the ethmoid cells and frontal sinus. Finally, the infection spreads through the posterior wall of the frontal sinus to the anterior cranial fossa. The occurrence of remote intracranial infection (e.g., intracerebral abscess, epidural abscess, subdural empyema) is possible due to the venous drainage of the face and paranasal cavities into the cerebral venous sinuses.

ASBO can also be iatrogenic, for example, after endoscopic surgery of the nose and paranasal sinuses [9]. The occurrence of SBO after bilateral transnasal endoscopic sphenopalatine artery cauterisation and sphenoidotomy has been described [10]. The occurrence of clival abscess or osteomyelitis after adenoidectomy [11,12] and epipharyngeal cyst excision [13] has also been described. Thornwaldt’s cyst infection can also cause ASBO [14]. Injuries to the skull base, especially in the case of contaminated wounds (e.g., sharp or gunshot injuries), can lead to ASBO [5]. In patients after head and neck cancer radiotherapy, the tissue at the skull base is more susceptible to osteoradionecrosis and SBO [15].

In some cases, the cause of ASBO is unknown. Some authors also report otogenic ASBO without otoscopic signs of ear infection. The absence of signs is associated with regression of necrotising external otitis, but the progression of infection at the skull base spreads to the clivus [6,16].

### 2.2. Patients’ Predispositions

Mostly, infection usually does not progress to ASBO, as certain patient predispositions or virulence factors of the microbe are also required [11,17]. Patients with diabetes and immune suppression are particularly susceptible [18]. Diabetes causes immunodeficiency and poor blood circulation due to damage to small (i.e., microangiopathy) and large vessels (i.e., macroangiopathy), which hampers tissue regeneration. Similarly, other diseases that cause vascular damage or poor oxygen delivery, e.g., post-irradiation changes, vasculitis, cancer, diseases of bone metabolism (e.g., osteoporosis, osteopetrosis, Paget’s disease), malnutrition, anaemia, cardiovascular diseases, liver failure, kidney failure, smoking, obesity, chronic lung disease, and prolonged hospitalisation, predispose to the development of SBO [4,19]. Advanced age is an independent risk factor [5]. Risk factors for ASBO are rarely absent [4,20]. For that reason, they must be identified and controlled.

### 2.3. Causative Microbes of Atypical Skull-Base Osteomyelitis

ASBO is most commonly caused by *Staphylococcus aureus*, followed by *Pseudomonas aeruginosa* and atypical mycobacteria. *Pseudomonas aeruginosa* is less likely to cause ASBO than typical SBO. More commonly than typical SBO, ASBO results from a fungal infection, most commonly with *Aspergillus* spp., usually in patients with neutropenia. Less often, ASBO results from infection with *Candida* spp. [5], *Rhizopus* spp., and *Mucor* spp., the latter usually in patients with diabetic ketoacidosis, causing a clinical picture of acute fulminant invasive fungal rhinosinusitis [4,18,19,21]. In the literature, cases of ASBO due to infection with the *Streptococcus anginosus* group, *Eikenella corrodens*, *Serratia marcescens*, *Enterococcus faecium*, *Peptostreptococcus* spp., *Mycobacterium tuberculosis* [5,8], *Klebsiella pneumoniae* [15], *Propionibacterium acnes* [10], *Nocardia* spp. [22], *Morganella* spp. [23], and *Bacteroides* spp. [5] are described.

More often than in typical SBO, the results of microbiological tests are negative in ASBO, making the diagnosis even more complex [9].

## 3. Epidemiology of Atypical Skull-Base Osteomyelitis

The frequency of ASBO is decreasing, most likely due to the development of diagnostics and treatment, especially antimicrobials. The greater frequency of this disease is seen in less developed and developing countries. However, the frequency of ASBO is unknown, as there is a lack of epidemiological research. The available literature on ASBO consists mainly of case presentations with literature reviews and monocentric research publications. For example, in a study by Johnson et al. (2014), which reviewed the literature on ASBO published in databases between 1946 and 2014, only 42 cases were included in the analysis, confirming that the disease rarely occurs [18]. Scoping reviews and then probably systematic literature reviews with meta-analyses would be needed.

## 4. Clinical Picture and Diagnosis of Atypical Skull-Base Osteomyelitis

ASBO usually presents with nonspecific symptoms, such as headache and facial pain. At the same time, other symptoms are usually present as part of an infection of the nose and paranasal sinuses (e.g., a clinical picture of rhinosinusitis), face (e.g., cellulitis or furuncle), oral cavity (e.g., odontogenic abscess), or pharynx (e.g., pharyngitis), which is confirmed by clinical examination. Otalgia may be present, but without otoscopic signs of ear infection, as then SBO would be diagnosed as typical (i.e., otogenic). Fever is present in less than 20% [4]. ASBO should always be considered in a patient with an infection in the ENT (ear, nose, and throat) area [6,18,19] when there are signs and symptoms of concomitant cranial nerve malfunction, especially of VIth and lower cranial nerves (mainly IXth and Xth), disturbances of consciousness, and other neurological deficits. Impairment of cranial nerve function is present in approximately half of the cases of ASBO [18]. Damage of the VIIth cranial nerve, manifested by facial paresis, is present in 21%, although it is more characteristic of typical SBO [18]. The role of the otorhinolaryngologist is essential in performing a thorough clinical examination, which also includes an examination of the cranial nerve function and tests of hearing and balance if the patient’s health condition allows it.

An untreated disease leads to intracranial complications, e.g., meningitis, epidural abscess, subdural empyema, intraparenchymal brain abscess, and subarachnoid haemorrhage. In addition, the infection can cause the formation of a mycotic aneurysm of the internal carotid artery, which, if ruptured, can lead to death from epistaxis or stroke [19,24]. An otorhinolaryngological examination is not sufficient to diagnose ASBO, but it is crucial to perform it to establish suspicion and carry out the appropriate diagnostic methods. Therefore, any patient suspected of having ASBO should undergo imaging and pathological and microbiological examinations of the tissue samples described below as soon as possible.

A summary of the main characteristics of ASBO is depicted in Table 1.

### 4.1. Imaging Studies

Due to nonspecific clinical features and laboratory findings, radiological evaluation plays a prominent role in establishing a diagnosis in cases of ASBO. Using different imaging modalities enables early diagnosis of ASBO and establishes the disease’s location and extent [27]. Generally, a combination of computerised tomography (CT, with or without contrast enhancement) and contrast-enhanced magnetic resonance imaging (MRI) is used to diagnose and monitor ASBO. However, MRI and CT findings often lag behind clinical improvement [28,29,30]. In exceptional cases, nuclear medicine studies can provide complementary information to help localise, differentiate the disease, and monitor the treatment response [31,32,33,34,35,36,37]. Communication between the otolaryngologist, neurosurgeon, and neuroradiologist is crucial in interpreting the results of these tests, as the neuroradiologist can also answer specific questions.

Imaging abnormalities of bone marrow and affected soft tissues in ASBO and typical SBO are essentially the same. However, by definition, ASBO is non-otogenic in origin and thus has a predilection for involvement of the central skull base, especially the clivus. In addition, greater and lesser sphenoid wings and petrous apices can also be involved. In contrast, in typical SBO, a lateral skull base, especially the temporal bone and the structures neighbouring the external auditory canal, are affected [6,18,38].

In addition to direct signs of SBO, imaging can also reveal the origin of the infection: iatrogenic or traumatic injury, infection in the oral cavity, nose, paranasal sinuses, or in the soft tissues of the face or scalp [39,40,41].

#### 4.1.1. CT Imaging

**Figure 1:**
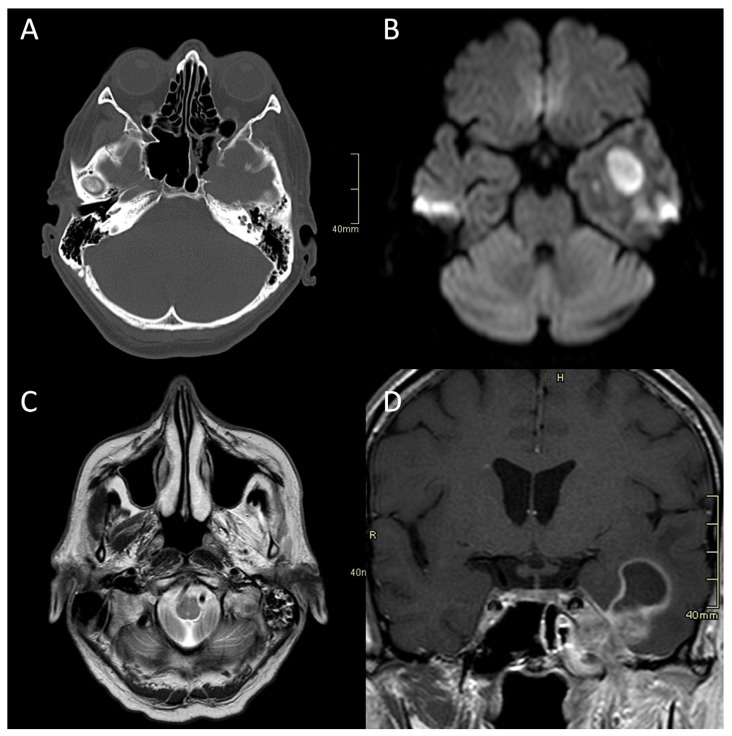
CT and MRI of a patient with fungal atypical skull-base osteomyelitis due to acute invasive fungal sphenoid sinusitis. (**A**) Axial CT bone algorithm reconstruction showing erosion of the lateral wall of the sphenoid sinus, indicating the spread into the cavernous sinus and middle cranial fossa toward the Meckel’s cave. (**B**) Axial MRI DWI showing increased signal in the left temporal lobe. (**C**) Axial MRI T2 showing increased signal in left masticatory muscles due to the denervation corresponding to the damage of the trigeminal nerve. (**D**) Coronal CE MRI T1 showing the spread of the infection from the sphenoid sinus into the parasellar space, cavernous sinus, and Meckel’s cave, with an intraparenchymal abscess of the temporal lobe. CT: computerised tomography; MRI: magnetic resonance imaging; DWI: diffusion-weighted imaging; CE: contrast-enhanced.

**Figure 2:**
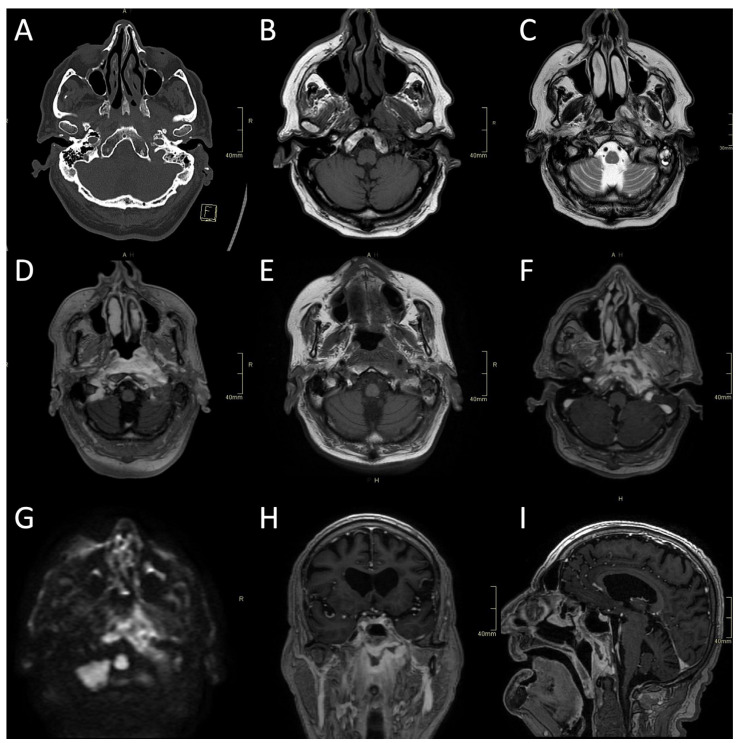
CT and MRI of a fungal atypical skull-base osteomyelitis patient. (**A**) Axial CT bone algorithm reconstruction showing subtle cortical erosion of the left skull base and the left carotid canal; F—foot. (**B**) Axial MRI T1 showing subtle asymmetrical hypo-intensity, indicating oedema of the bone marrow on the left side of the clivus. (**C**) Axial MRI T2 showing asymmetrical hyperintensity in pharyngeal soft tissues and prevertebral muscles, indicating oedema and inflammation. (**D**) Axial MRI T1 showing the progression of oedema of the bone marrow of the clivus six weeks later. (**E**) Axial CE MRI showing the progression of hyperintensity in pharyngeal soft tissues and prevertebral muscles and affected bone marrow. (**F**,**H**,**I**) Axial, sagittal, and coronal CE MRI T1 showing extensive inflammatory changes in skull-base bone marrow and soft tissues, with non-enhancing branching fluid collection—abscess in the pre-clival and para/retropharyngeal soft tissues after 11 weeks. (**G**) Axial MRI DWI showing increased signal in the central skull base. CT: computerised tomography; MRI: magnetic resonance imaging; CE: contrast-enhanced; DWI: diffusion-weighted imaging.

**Figure 3:**
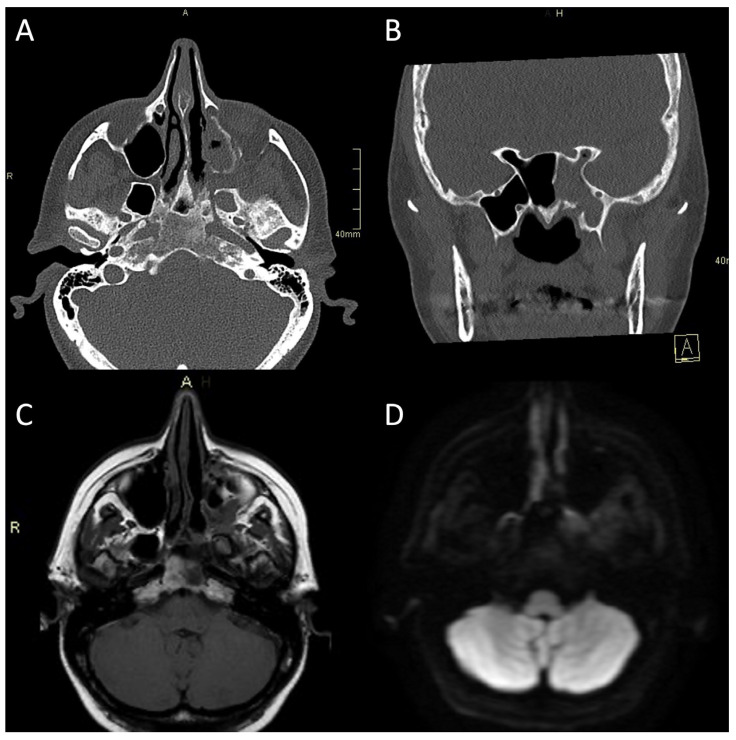
CT and MRI of a patient with bacterial atypical skull-base osteomyelitis. (**A**) Axial CT bone algorithm reconstruction showing expanded left pterygopalatine fossa, opacified ipsilateral maxillary, and sphenoid sinus, with mixed erosive and hyperostotic bony changes. (**B**) Coronal CT bone algorithm reconstruction showing asymmetric expansion and destruction of the left Vidian canal; A—anterior. (**C**) Axial MRI T1 showing clival bone marrow oedema. (**D**) Axial MRI DWI showing increased signal in the left pterygoid base, pterygopalatine fossa, and infratemporal fossa. CT: computerised tomography; MRI: magnetic resonance imaging; CE: contrast-enhanced; DWI: diffusion-weighted imaging.

CT is often the first imaging modality in patients with SBO as it is the most accessible and can be performed in the urgent setting. In addition, CT, especially high-resolution bone reformats, is the best way to assess the often subtle bone erosion and demineralisation characteristic of SBO [4,9,18,42]. Cortical erosions in ASBO are most often noted along the anterior clivus and foramina of the central skull base; in cases of invasive sinusitis, opacification and erosion of sinus walls can be noted, especially sphenoid sinus and ethmoid cells (Figure 1A, Figure 2A and Figure 3A,B) [6].

Contrast-enhanced CT can show changes signifying phlegmon (obliteration of fat planes, ill-defined contrast enhancement, tissue fullness) or a frank abscess of affected soft tissues [4,19]. In case of vascular complications, CT angiography (CTA) or CT venography (CTV) can be performed to determine vessel patency.

#### 4.1.2. MR Imaging

MRI is superior to CT in depicting the extent and presence of bone marrow involvement, soft tissue changes, and possible intracranial complications [38]. Therefore, the MRI protocol should include at least T1, T2, STIR (short tau inversion recovery), DWI (diffusion-weighted imaging), and T1 FS (fat-saturated) images after contrast injection in different planes according to the local anatomy.

**Figure 4:**
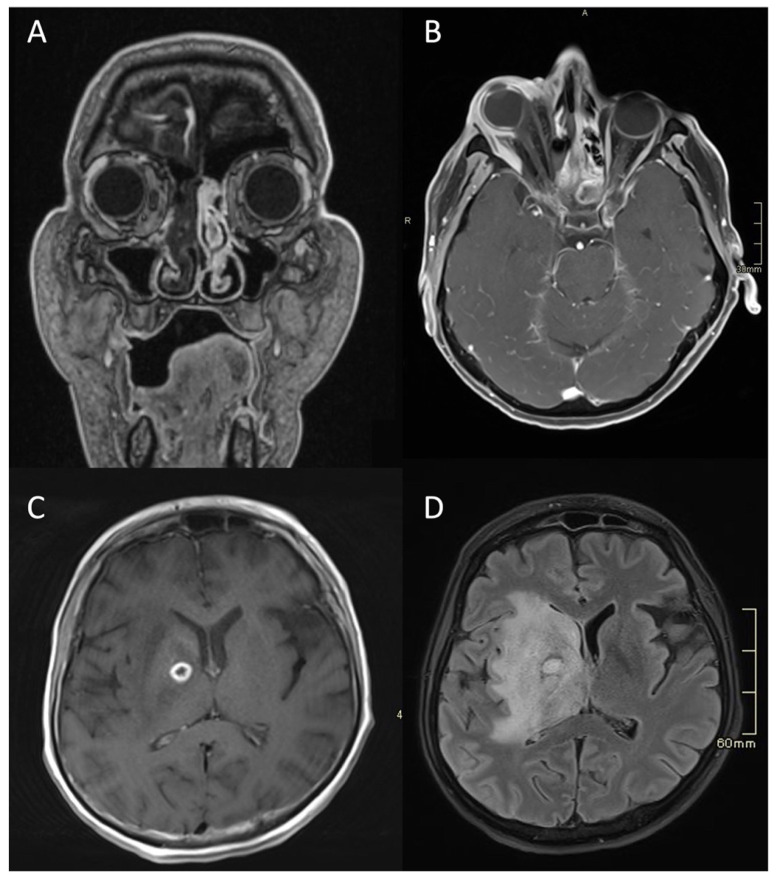
MRI of a patient with an acute fulminant invasive fungal atypical skull-base osteomyelitis due to rhino-orbital-cerebral mucormycosis. (**A**) First coronal CE MRI T1 showing a lack of a contrast enhancement in the right nasal cavity and paranasal sinuses, indicating necrosis in the context of invasive mucormycotic infection (i.e., black turbinate sign). (**B**) Axial CE MRI T1 FS two weeks later, showing significant right-sided proptosis, signs of increased intra-orbital pressure, a lack of enhancement in the lateral and partially in the medial rectus muscle, and enhancing and non-enhancing parts of the eye bulb—signs of inflammation and necrosis. (**C**) Axial CE MRI T1 showing an abscess, which is a ring-enhancing lesion in the brain, with signs of restricted diffusion on DWI (not shown). (**D**) Axial flair MRI showing extensive oedema and abscess in the deep structures of the right hemisphere three months later. MRI: magnetic resonance imaging; CE: contrast-enhanced; FS: fat-saturation; DWI: diffusion-weighted imaging.

Typical for ASBO is symmetric pre-clival soft tissue involvement, producing nasopharyngeal swelling (Figure 2D,E). In addition, invasive sinus infections have a predilection for spread in the pterygopalatine fossa (Figure 3C,D) [4]. The infection of bone marrow in ASBO results in a loss of normal fat signal, which is seen as T1 hypo-intensity (Figure 2B) and STIR hyperintensity, with heterogenous contrast enhancement of the affected marrow [43,44]. Peripheral contrast enhancement is seen when an abscess is formed in the necrotic part of the bone marrow (Figure 2F,H,I). A combination of abnormal enhancement and absence of contrast enhancement is observed, particularly in mucormycotic infection, indicating the presence of inflammation and necrosis (Figure 4A,B) [45].

Imaging studies, both MRI and CT, including CTA and CTV, are essential in diagnosing complications of SBO. These include the intracranial spread of infection, the formation of an abscess, either intracranially (Figure 1B,D and Figure 4C,D) or in the soft tissues of the head and neck (Figure 2F,H,I), and vascular complications—venous thrombosis, venous or arterial stroke, and mycotic aneurism formation [30,46,47].

From the radiological viewpoint, the principal differential diagnosis for ASBO is a neoplastic disease. Several studies have noted that the apparent diffusion coefficient (ADC) values in patients with SBO are higher than those with neoplastic disease, which is the main differentiator [48,49]. Other imaging features favouring neoplastic diseases are a dominant soft tissue mass or a nodule with secondary bone involvement, architectural distortion with displacement or destruction of normal tissue planes, and enlarged neck lymph nodes [9,50,51,52].

#### 4.1.3. Nuclear Medicine Studies

With the advent of MRI and CT, the role of nuclear imaging in evaluating SBO declined [31]. However, nuclear imaging can still help to confirm and localise bone involvement and help monitor the response treatment in special cases. For example, technetium methylene diphosphonate (99mTc MDP) scintigraphy, single-photon emission CT (SPECT), gallium-67 (Ga-67) scintigraphy, indium–111 (In-111) oxine-labelled white blood cell scan, or fluorodeoxyglucose-positron emission tomography (FDG PET) can be performed and show sites of increased bone turnover, inflammation, and increased metabolism in SBO [31,33,53,54,55]. Amino acid PET tracers have an emergent role in differentiating brain tumours from non-neoplastic pathologies such as ASBO [56].

### 4.2. Otorhinolaryngological Examination and Tissue Specimen Sampling

**Figure 5:**
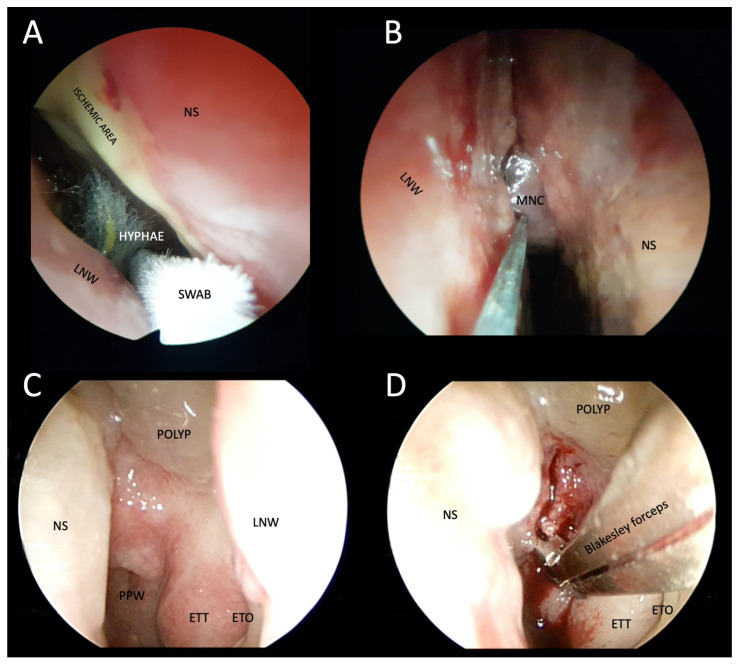
Photographs of transnasal endoscopic collection of pus and tissue specimens for microbiological and histopathological examination in patients with suspected atypical skull-base osteomyelitis. (**A**,**B**) Swabs were taken from a patient with rhino-orbital-cerebral mucormycosis. Fungal hyphae are visible in the right nasal cavity, ischemia of the nasal septum, and the middle nasal concha necrosis, which has a blackish appearance (i.e., black turbinate sign). (**C**) Thickened epipharyngeal tissue with normal mucosa in a patient with atypical fungal skull-base osteomyelitis. (**D**) Deep biopsy after epipharyngeal incision. LNW: lateral nasal wall; NS: nasal septum; MNS: middle nasal concha; PPW: posterior pharyngeal wall; ETT: eustachian tube torus; ETO: eustachian tube orifice.

Representative samples must be taken for pathological and microbiological examinations (Figure 5).

**Figure 6:**
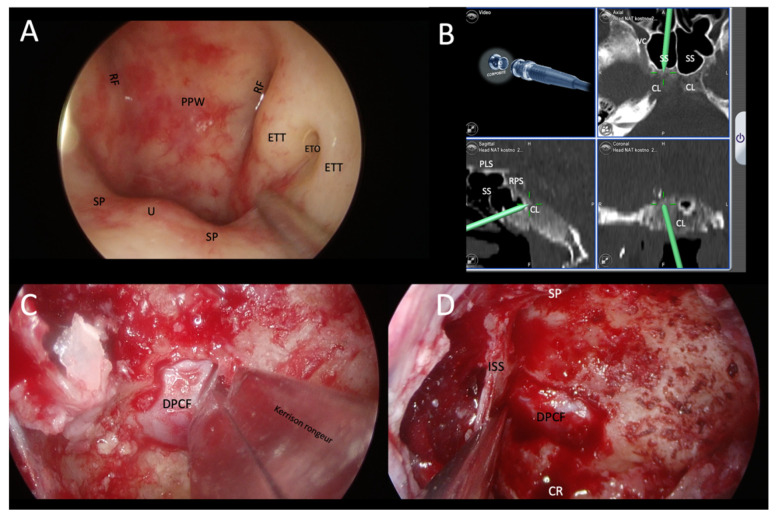
Demonstration of transnasal transsphenoidal endoscopic biopsy of a skull-base lesion using image guidance in a patient with EBV-positive squamous cell carcinoma of the epipharynx. (**A**) Normal epipharynx anatomy. It was necessary to perform an extended sphenoidotomy with a transclival biopsy, in which the lesion was identified by image guidance. (**B**) Identification of a hypodense lesion in the mid-clivus below the sella turcica. (**C**,**D**) Showing the surgical procedure of sphenoidotomy and removal of the posterior wall of the sphenoid sinus to the dura of the posterior cranial cavity. Bone fragments were sent for histopathological examinations. RF: Rösenmuller’s fossa; PPW: posterior pharyngeal wall; ETT: eustachian tube torus; ETO: eustachian tube orifice; SP: soft palate; U: uvula; CL: clivus; VC: Vidian canal; SS: sphenoid sinus; PLS: planum sphenoidale; RPS: right parasellar space; DPCF: dura of posterior cranial fossa; SP: sellar prominence; ISS: intrasphenoid sinus septum; CR: clival recess.

In general, we strive for the least invasive method of tissue biopsy, as enabled by the transnasal endoscopic technique and image guidance (Figure 6).

A good collaboration between the otorhinolaryngologist and the neurosurgeon is necessary during the biopsy, especially in patients with a high risk of intracranial complications during the biopsy. In addition, it is necessary to know the correct protocols for storing and transporting samples for further investigations.

In case of suspicion of an intracranial infection (e.g., meningitis), in the absence of contraindications, a lumbar puncture and cytological and biochemical tests of the cerebrospinal fluid are performed.

### 4.3. Histopathology

Histopathologic examination in patients with osteomyelitis is an essential diagnostic tool that enables characterising morphologic features of inflammation, searching for microorganisms, and excluding other diseases, such as tumours and vasculitis. Histopathologic features in atypical osteomyelitis are variable and depend on the causative agent and duration of the disease, as well as on the patient’s comorbidity and immune status. They include acute and chronic inflammatory cell infiltration, granulomas, necrosis, oedema, the proliferation of granulation tissue, and fibrosis. In addition, histopathology may help to determine the aetiology; for example, mycobacterial or mycotic infection if granulomatous inflammation is present, fungal infection in cases with abundant necrosis and vascular invasion, and viral infection in cases with either nuclear or cytoplasmatic inclusions. Histopathologic features may, however, be entirely nonspecific, particularly in bacterial infection.

Special staining techniques must be used to search for microorganisms. They include silver impregnation stains (Grocott or Gomori methenamine silver) and periodic acid-Schiff (PAS) for fungal infection, Gram staining for bacteria, and Ziehl–Neelsen staining and Auramin-rhodamin for mycobacteria [57]. In addition, in situ hybridisation and polymerase chain reaction (PCR)-based methods may be used but are unavailable for all microorganisms [57,58].

The histopathologic examination may play a crucial role in diagnosing fungal, mycobacterial, and viral infections, but has a very limited, if any, role in bacterial infections, with one exception, i.e., actinomycotic infection. Actinomycosis can be recognised in tissues by colonies of filamentous bacteria that appear as round basophilic masses with a radiating arrangement of eosinophilic terminal “clubs”. Special stains are helpful to confirm the diagnosis, as actinomyces stain with silver impregnation techniques and are Gram-positive. For microbiologic diagnosis of actinomycosis, anaerobic culture is required. It has been claimed that culture results in a proper diagnosis in only 20% of cases, whereas the remaining cases are diagnosed by biopsy. The biopsy is considered the quickest and most sensitive way of diagnosing actinomycosis [59,60].

One of the most critical tasks of histopathology is to provide a rapid diagnosis of mycotic infection while waiting for culture results, or it may even be the only available material when no culture growth occurs, or cultures are not ordered. Morphological analysis may suggest the type of mycotic agent. *Aspergillus* spp. is characterised by acute-angle-branching septated hyphae. Recognition of *Mucor* spp. is based on identifying broad, ribbon-like pauciseptate hyphae, with right-angle branching [61]. The pseudo-hyphae of *Candida* spp. are thin, regular, and without true septation. Microbiologic and molecular techniques must be used to reliably identify the fungal genus and species [61,62,63].

### 4.4. Microbiology Studies

Due to the difficult accessibility to the site of the affected tissues and the initial hidden, nonspecific clinical picture, the diagnosis is difficult and complex. Early clinical suspicion of ASBO is, therefore, crucial. In the next phase, the clinical microbiologist defines the aetiology of the disease, which allows the introduction of targeted antimicrobial treatment. Classic microbiological methods include microscopy and cultivation. The gold standard of microbiological diagnostics is cultivating the causative agent on appropriate microbiological culture media. Isolation of the causative agent from primarily sterile specimens (most often a biopsy of inflamed tissue) is proof and confirmation of invasive infection. Sterile sampling is rarely performed in ASBO since the specimens are collected transnasally. Therefore, it is optimal that the collection is carried out before initiating antimicrobial treatment. The inflamed, changed tissue is aseptically removed into a sterile container, to which two drops of saline are added to prevent specimen drying. Obtaining three to five pieces of tissue (minimum 50 mg) is recommended. The samples are delivered to the laboratory two hours after collection. Although transport usually takes place at room temperature, the tissue must not dry out because, in the presence of the fungi of the order Mucorales, they can collapse and perish.

If, during the sampling with the sampling instruments, we pass the areas that are inhabited by normal microbiota, as it is most often in ASBO, the interpretation of the results of the microbiological tests is more complex, since many of the microorganisms that are isolated with the sampling obtained in this way may represent the normal permanent or transient microbiota of the upper respiratory tract. They, however, can be the causative agents of infection at the same time. In this case, histopathological findings can resolve the dilemma, which confirms the presence of microorganisms and the inflammatory reaction in the histological sections.

If the transported tissue is sufficient, part of it is prepared for direct staining. First, bacteria are visualised by Gram staining. Then, the same preparation can be stained in the second phase with a fluorescent dye that binds keratin (usually calcofluor white), with which fungal structures can be visualised with an ultraviolet microscope. In the case of the presence of *Aspergillus* spp., we observe narrow (3–10 µm), dichotomously branched hyphae with uniformly distributed hyphae, while in the fungi of the order Mucorales, we see wide (10–20 µm) ribbon hyphae, which are sparsely, unevenly septate and split at right angles [64,65].

Fluorescent methods (e.g., auramine staining) are usually used to visualise mycobacteria in direct microscopic preparation. The fast-growing bacteria and fungi that are usually the causative agents of ASBO on enriched media usually grow after 24–48 h. Identification is carried out by MALDI TOF (matrix-assisted laser desorption-ionisation-time of flight) mass spectrometry, and in the case of fungi, we also use morphological identification of the causative agents. If we cannot identify the causative agent with these methods, we use molecular methods, and identification by sequencing. Nontuberculous mycobacteria grow more slowly. Therefore, we identify them by their morphological characteristics, pigment, and molecular methods. Molecular tests are also used to directly detect causative agents of nontuberculous mycobacteria from tissue [66,67,68].

The growth of microorganisms can be delayed if the tissue is less oxygenated, or if the patient receives antimicrobial treatment. In the latter, growth may also be absent. Molecular tests complement standard microbiological diagnostics, as they are distinguished by a higher sensitivity and specificity. In the case of suspicion of ASBO, it is necessary to make a pragmatic decision on the choice of tests in cases where we do not have enough samples for all available tests. When the patient is immunodeficient and possibly has advanced diabetes and other known risk factors for developing a fungal infection, we often think of mucormycosis or invasive aspergillosis. We have commercially available kits that detect the presence of nucleic acids from the order Mucorales and multiplex PCR for detecting nucleic acids of *Aspergillus* spp. [64,65]. The tissue usually needs to be enzymatically treated before the molecular reaction, which prolongs the diagnosis by at least 24 h. Molecular methods are also used to detect the most common bacterial pathogens. Specific multiplex PCR for individual or a few of the most common pathogens and nonspecific eubacterial PCR that detect the entire spectrum of bacterial pathogens are available. The former is characterised by a higher sensitivity, while the latter is nonspecific, with a lower sensitivity. Sometimes, we encounter the dilemma of whether the result is the result of contamination or whether it is an indication of the actual causative agent of the disease.

A multidisciplinary approach combining classic and molecular microbiological examinations with histopathological examinations can thus help define the aetiology, which facilitates interpretation and increases the accuracy of diagnosis. Molecular methods of direct detection of the causative agents are also performed from fresh tissue obtained by biopsy. The causative agent can also be identified in exceptional cases from formalin-fixed paraffin-embedded tissues (FFPET). This procedure is used mainly in cases where fungal structures are observed in pathohistological tissue sections, but mycological cultures have no growth. The same applies to bacterial infections. In these cases, it is imperative to carefully interpret negative results because they can be false negatives due to DNA fragmentation during isolation from FFPET.

### 4.5. Blood Tests

Laboratory blood tests can determine the presence of systemic inflammation or infection before imaging diagnostic methods and pathological and microbiological examinations are carried out. For ASBO, the blood concentrations of C-reactive protein and leukocytes are usually not elevated as markedly as the erythrocyte sedimentation rate (ESR) [4,6]. Moreover, obtaining blood cultures is reasonable if the patient has general inflammation symptoms.

Determining fungal antigens in the blood is also used in diagnosing ASBO. Among them, the determination of the nonspecific biological marker β-d-glucan and, in the case of suspected invasive aspergillosis, the determination of galactomannan, may be helpful. Nevertheless, it should be emphasised that it is necessary to correctly interpret the results and know the factors for false negative and false positive results [69].

## 5. Differential Diagnosis of Atypical Skull-Base Osteomyelitis

Due to the non-characteristic clinical picture and radiological examinations, ASBO shares characteristics with other skull-base diseases, so the diagnosis can be established too late. The disease must often be differentiated from typical SBO, tumours, and rheumatological diseases [18].

### 5.1. Typical (i.e., Otogenic) Skull-Base Osteomyelitis and Necrotising External Otitis

Typical SBO is a complication of necrotising external otitis or chronic mastoid inflammation. Mainly, necrotising external otitis is caused by *Pseudomonas aeruginosa*, which is the causative microbe of typical SBO in 90–98% of cases [25,26].

Despite new diagnostic methods and numerous studies, the exact pathogenesis of typical SBO remains unknown. It typically affects elderly patients with diabetes or a deficient immune system. In these patients, the alkaline pH of the cerumen may lead to a weakened immune response due to an impaired phagocytosis and leukocyte response. At the same time, patients with necrotising external otitis often have microangiopathy and hypoperfusion, which increases the risk of osteomyelitis. The aural toilet also increases susceptibility to *Pseudomonas aeruginosa* infection in elderly diabetic patients, as the external ear canal becomes less acidic and contains less lysozyme [70].

Necrotising external otitis can spread from the external auditory canal to the skull base through the fissures of Santorini, which lie on the inferior wall of the external auditory canal, and through the bone–cartilaginous junction of the external auditory canal [70]. This progressive spread of the infection to the foramina of the skull base can damage the cranial nerves, most often the facial nerve. Anteroinferior spread of infection leads to parotitis and temporomandibular joint arthritis. Inferomedially, the inflammation may extend to the internal carotid artery and jugular bulb. Posteriorly, the infection can spread into the sigmoid sinus and medially deep into the clivus, leading to a misdiagnosis of ASBO [71].

Diagnosing typical SBO is based on specific findings from the history, clinical examination, laboratory blood tests, imaging, and microbiological and histopathological examinations. This disease is treated with broad-spectrum antimicrobials, which can then be replaced with narrow-spectrum antimicrobials depending on the result of the antibiogram. In widespread soft tissue involvement, early surgical removal of infected sequestrations is required [72].

### 5.2. Rheumatological Diseases

The rheumatological diseases most often considered in the differential diagnosis of ASBO are granulomatosis with polyangiitis (formerly Wegener’s granulomatosis) and IgG4-related disease. Although both are systemic diseases, immune suppression is the basis of treatment for both. The prognosis of the disease at the skull base is then good.

Granulomatosis with polyangiitis is an idiopathic vasculitis of small- and medium-sized vessels. It usually occurs as a triad of renal, pulmonary impairment, and epipharyngeal involvement. Symptoms due to the involvement of ENT areas present first in 80% of cases at the beginning of the disease. These patients can suffer from ASBO [73].

The IgG4-related disease, first described in 2001, is also a systemic disease that can manifest itself with symptoms of involvement in ENT areas. It is characterised by an extensive infiltration of IgG4-positive plasma cells and T-cells, which causes the formation of tumour-like lesions. Several manifestations of this disease are known, including osteomyelitis-like lesions at the skull base, Küttner’s tumour of the submandibular salivary gland, Hashimoto’s thyroiditis, and Riedl’s thyroiditis [74].

### 5.3. Tumours

Malignant tumours of the skull base are better known than ASBO, so they usually do not present a differential diagnostic challenge. We often encounter nasopharyngeal squamous cell carcinoma (Figure 6) and, less often, other histological types of tumours and metastases at the skull base [18,19]. History is essential in distinguishing ASBO from a tumour.

Benign tumours of the skull base occur less often than malignant tumours, but clinical differentiation can be challenging, especially in well-perfused benign tumours. Differentiating benign tumours from ASBO is usually less challenging, as there are no systemic symptoms. In some cases, imaging is sufficient to establish the diagnosis, which is the case for fibrous dysplasia [19].

## 6. Treatment of Atypical Skull-Base Osteomyelitis

Patients with ASBO are usually treated in the department of infectious diseases because of long-term antimicrobial therapy and poor general health. The infectious disease specialist leads the treatment and includes other specialists, including—an otorhinolaryngologist, neurosurgeon, radiologist, clinical microbiologist, and a pathologist. The mainstay of treatment for ASBO is antimicrobial therapy [18]. At the same time, managing all risk factors for developing ASBO is necessary, e.g., treating diabetes and immune deficiency [19]. 

### 6.1. Surgery

**Figure 7:**
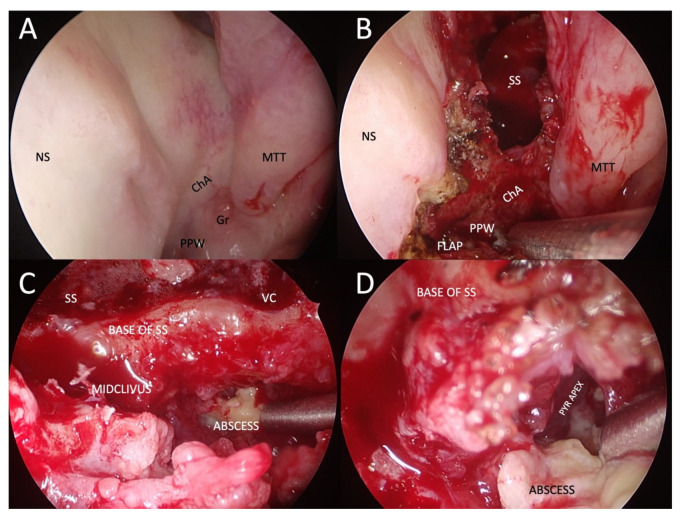
Example of transnasal endoscopic drainage of clival abscess in a patient with left-sided fungal atypical skull-base osteomyelitis. (**A**) Nasal endoscopy after decongestion, showing very subtle abnormalities, only a granulation tissue on the left side of the nasopharynx and a poorly defined choanal arch. (**B**) Paraseptal left-sided sphenoidotomy and elevation of nasopharyngeal flap pedicled on the inferomedial posterior nasopharyngeal wall. (**C**) Close-up view of the base of the left sphenoid sinus and mid-clivus after drilling to gain access to the abscess cavity. (**D**) Abscess cavity is drained with blunt dissection to visualise the tract under the pyramid apex. NS: nasal septum; ChA: choanal arch; MTT: middle turbinate tail; PPW: posterior pharyngeal wall; SS: sphenoid sinus; VC: an area of the Vidian canal; PYR APEX: pyramid apex.

Surgical treatment has a role in ASBO as a complement to antimicrobial treatment. Often, it would not be possible to eradicate the disease surgically, or the procedure would be too risky. At the same time, patients are more susceptible to the surgical risks of extensive skull base surgery due to their generally poorer health status and proximity of the vital neurovascular structures (e.g., internal carotid artery, basilar artery, lower cranial nerves). The purpose of surgical treatment is tissue biopsy, decompression of vital neurovascular structures (e.g., transnasal endoscopic transodontoid decompression of the brainstem, optic nerve decompression), abscess drainage (Figure 7), drainage of the paranasal cavities, partial necrectomy, or sequestrectomy, to reduce the microbial load, to improve tissue perfusion, and consequently, for better penetration of antimicrobials. Nevertheless, indications for surgical treatment are still unclear [18,42,75].

### 6.2. Antimicrobial Treatment

Long-term pathogen-specific antimicrobial therapy remains the mainstay of treatment [18]. Early empiric therapy with broad-spectrum intravenous antibiotics should include coverage for *Pseudomonas aeruginosa* (e.g., antipseudomonal beta-lactam, a third-generation cephalosporin or carbapenem) and methicillin-resistant *Staphylococcus aureus* (e.g., vancomycin) [4,9,42]. A biopsy should be performed before initiating antimicrobial therapy to increase the microbiologic yield. Unless the patient’s history, microbiological (culture/fungal markers), or pathology results suggest fungal infection, empiric broad-spectrum antifungal therapy is not indicated. However, it should be considered if there is no clinical improvement despite appropriate empiric antibiotics [15,18]. According to the literature, the optimal treatment duration is unknown. Based on case reports and case series, the suggested length of antimicrobial therapy is 6–20 weeks [4,9,18,42], started by intravenous antimicrobial therapy for a minimum of 6 weeks [9,18]. The final duration of antimicrobial therapy with a good treatment response should be based on clinical status and serial inflammatory markers, whereby a normalisation of ESR is a good indicator of infection resolution [6,18]. Long-term monitoring with CT or MRI is generally not helpful because radiologic abnormalities of the bone may persist for weeks to months despite clinical improvement [4,18]. However, radiologic imaging should be performed in case of clinical deterioration. The variable duration of treatment is highly based on the patient’s immune status, the extent of the initial infection, the opportunity for source control procedures, the tolerability of antimicrobial therapy, and the risk of treatment failure [15]. Nevertheless, there is no evidence of an association between diabetes and the longer duration of antimicrobial therapy [18]. In patients with fungal infection (especially in immunocompromised patients), oral antifungal therapy is usually prolonged by up to 6–12 months, or even more, and depends on the patient’s underlying disease, immune status, and response to therapy.

After stopping the antimicrobial therapy, careful follow-up with close monitoring of the patient’s clinical symptoms is needed [15,18]. Based on the case series, there is a significant risk of recurrent infection. For example, in a systematic review by Johnson and Batra (2014), the average patient required 1.3 courses of therapy because of relapse or recurrence of symptoms. In contrast, in a retrospective study of head and neck cancer patients with skull-base osteomyelitis conducted by Czech et al. (2022), the recurrence was even higher, with nearly half of the patients having multiple episodes of recurrent infections [15,18].

### 6.3. Hyperbaric Oxygen Therapy

Hyperbaric oxygen therapy is effective in some cases of ASBO. However, the research is insufficient, so routine use is not indicated. Nevertheless, this method has a role as a complementary treatment, especially in recalcitrant ASBO [5,18]. This treatment increases the partial pressure of oxygen in tissues, reduces tissue hypoxia, improves phagocytosis, and accelerates angiogenesis and osteogenesis. An example of a hyperbaric oxygen treatment regimen is a 90-min dive at a pressure of 2.5 atmospheres, 5 days a week, for 1 month [19].

## 7. Prognosis of Atypical Skull-Base Osteomyelitis

ASBO is a serious life-threatening condition with the possibility of severe complications. It presents with at least one cranial nerve dysfunction in 21–48% of cases. Although neurological improvement during treatment depends on the individual case and is not universal, paresis persists in approximately 30% of patients. Correction of paresis can occur due to nerve regeneration after a cured disease, decompression of neural structures, or compensation from the contralateral side. As such, surgical intervention does not directly contribute to improving neurological deficits. Post-operative deterioration may occur at the expense of radical resection of the affected bone, which surrounds the already damaged neural structures. The disease can be complicated by the spread of the infection to the surrounding soft tissues, and in rare cases, to the brain or meninges. Disseminated infection is associated with a higher risk of sepsis and increased mortality, despite surgical intervention and aggressive antimicrobial therapy [6,18,42].

Considering the relatively rare occurrence of the disease and the small set of studies, the predictive factors of the outcome of ASBO are not entirely clear. Associated diseases are generally the main prognostic factor, and worse disease outcomes have been described in elderly, male, diabetic, and immunocompromised patients, and patients with chronic ear disease [6,76]. The most important independent factor for a more favourable outcome is a multidisciplinary approach to the patient, with early and radical surgical removal of the focus of infection and adjuvant antimicrobial therapy. According to some studies, the treatment outcome is improved by the addition of hyperbaric oxygen therapy, which increases the partial pressure of oxygen and thereby reduces tissue hypoxia, and also improves phagocytosis and promotes angiogenesis and osteogenesis [19]. Clinical and radiological parameters, such as the resolution of the disease on imaging, the reduction of pain, and the improvement of cranial nerve paresis, speak in favour of a better outcome [42]. Other variables during treatment (e.g., duration of antibiotic therapy, number of repetitions of therapy) did not prove to be prognostically significant [18].

## 8. Conclusions

Atypical skull-base osteomyelitis is a rare, life-threatening, chronic infectious disease, with an uncharacteristic clinical picture. Knowing the pathogenesis of the disease, it is usually suspected in the elderly, patients with untreated chronic inflammation of the nose and paranasal cavities, and patients with known immune deficiencies, such as diabetes and blood diseases. Early diagnostic imaging, especially CT and MRI, interpreted by an experienced neuroradiologist, clinical examination with the collection of bacterial samples by a surgeon and infectious disease specialist, and examination of infectious agents by a pathologist and clinical microbiologist play a key role in diagnosis. It is fundamental to eliminate the cause of the immune deficiency and introduce antimicrobial drugs to cure the disease or halt its progression. Surgical treatment has a role in removing the focus of the disease within surgically safe confines and improving the effect of antimicrobial treatment.

## Figures and Tables

**Table 1:** Summary of the main characteristics of atypical skull-base osteomyelitis [4,18,25,26].

	Atypical Skull-Base Osteomyelitis	Typical Skull-Base Osteomyelitis
**Usual cause**	Advanced paranasal sinus infection	Necrotising external otitis
**Usual causative organism**	*Staphylococcus aureus*	*Pseudomonas aeruginosa*
**Other causative organisms**	More common fungi and other bacteria	*Pseudomonas aeruginosa* in 90–98%
**Usual microbiological tests results**	More often negative	More often positive
**Occurrence**	Very rare	Rare
**The usual initial clinical picture**	Nonspecific symptoms: headache and facial pain, the clinical picture of rhinosinusitis, facial cellulitis, furuncle, or pharyngitis	Headache, otalgia, aural discharge, conductive hearing loss, external auditory canal swelling, and granulation tissue
**Usually affected cranial nerves after**	VIth, IXth, Xth	VIIth

## Data Availability

Not applicable.
